# Five Cases of Removal of the Jaw for Tumor

**Published:** 1891-02

**Authors:** William D. Hamilton

**Affiliations:** Columbus, Ohio


### Five Cases of Removal of the Jaw for Tumor.

BY WILLIAM D. HAMILTON, M.D., OF COLUMBUS, OHIO.

Read in the section of Dental and Oral Surgery at the forty-first annual meeting of the American Medical Association at Nashville, Tenn., May, 1890.

The cases that form the subject of this paper have come under the observation of the writer in his service at the Mount Carmel Hospital within the past three years. Three had to do with the superior, and the remaining two with the inferior maxilla. There were four females and one male. The youngest patient was twelve, the eldest fifty-five years of age.

In conversation with the eminent surgeon, Mr. Thornley Stoker, of Dublin, last summer, it was learned that one of his patients for whom he had recently extirpated the upper jaw for sarcomatous disease, was a woman eighty years old. A still more interesting fact was elicited: that she was discharged from the hospital cured in a fortnight.

Cases of epithelioma beginning in the soft parts will not be herein considered. In this department of oral surgery we are confronted with certain facts at the outset: 1. Difficulty in maintaining anæsthesia. 2. The impracticability of observing with nicety antiseptic details. 3. The tendency to hemorrhage and shock.

It is to be hoped that rectal anæsthesia will some day be reduced to a practical method, so that an irritating fluid like ether may be prevented from entering the bowel in the liquid state, where it excites such great disturbance, or even perhaps a fatal bloody diarrhœa.

The renewal of the anæsthetic on account of the returning consciousness of a patient upon whom such an operation is being performed is a most embarrassing and pathetic necessity. It frequently means the occurrence of vomiting and the loss of invaluable time. The resort to a preliminary tracheotomy, while possessing certain advantages as a mode of administration, is by no means free from danger to the lungs and air passages which it is intended to protect, while its performance must add materially to the shock.

Unfortunately the mouth is frequently found to be in an unwholesome condition. The irregularities of the growth, which may prevent separation and closure of the jaws, the occasional existence of a foul ulcer that has been teased and irritated by opposing teeth, the numerous hiding places for septic matter in such a cavity—all these considerations make it a trap for filth, and enhance the difficulty of preventing suppuration. It is all the more incumbent upon the surgeon, however, to aim at thorough cleanliness. There should be liberal irrigations with a harmless antiseptic lotion like boracic acid in an aqueous solution, and in this way all removable matter disposed of in the five days prior to the operation.

The strict observance of extrinsic antiseptic details is as clearly imperative in this as in other surgical procedures.

As a preliminary step, with a view to preventing hemorrhage in removing some of these growths, the common or external carotid artery has been tied. While benefit may sometimes be derived from it, preparatory delagation was not seriously contemplated in any of the cases enumerated. In dealing with the facial artery and vein, the knife was carried quickly down to the bone dividing them clean at one stroke. Their prompt seizure with hæmostatic forceps prevents the loss of much blood, and simplifies the operation. Digital pressure by an assistant is available during their isolation.

With reference to shock it may be said that time is a most important element. In order to minimize the duration of the ordeal, scrupulous care should be taken to have a simple reliable armamentarium at hand consisting of strong instruments in perfect condition. For completing the division of bone a pair of powerful straight pliers, with short jaws and long handles, giving great leverage is invaluable.

For ligating vessels properly prepared silk is preferable. A precaution that should never be neglected is to transfix the tongue with a coarse silk thread; the ends being tied together should be held by an assistant. It is perhaps needless to observe that anæsthesia shold be *very profound* before beginning.

Again, it is a rule applying to all such undertakings to postpone entering the cavity of the mouth as long as it is consistent with method. Therefore the bone should be freed as extensively as may be before the mucous membrane is divided. In this way blood can often be kept outside of the throat until the operation is wellnigh completed.

Case 1.—Adeno-Sarcoma of Lower Jaw. Removal of left Half of Bone. Recovery.

Miss N. M., sent by Dr. A. M. Dent, of Coshocton, Ohio, was 27 years old when first seen by the writer. In July, 1884, she ran against the edge of a door in the dark and the left side of the face was struck. Some swelling followed which never receded. From the time of the injury until September, 1888, when she was admitted to the hospital, her face grew larger. The disease first appeared in front of the left masseter muscle and the contiguous portion of bone gradually became implicated. She stated that it had grown more rapidly since October, 1887, than during the preceding two years. A slight oral hemorrhage took place then, and pain had annoyed her most of the time since. The latter was attributable to the continual bruising of the tumor by the upper teeth. A surgeon having been consulted some months previously, had incised and scraped the prominent outer surface of the jaw, and with negative results. The month preceding the second operation had been of rapid progress of the growth. Painful sores existed where the upper teeth impinged upon it. The left side of the face had a rounded appearance, the cheek being prominently bulged outward. The mouth was encroached upon both posteriorly and toward the median line, so that it could only be opened for distance of seven-eighths of an inch. The ramus was extensively involved.

Operation September 30, 1888, Dr. F. W. Blake, and Dr. A. N. Dennison assisted. The usual incision was made and extended in the line of the old cicatrix. A clear exposure of the bone thus being secured, the dissection was completed with guarded scissors, care being taken to cut *on* the tumor throughout. While detaching it from the cheek, several sub-cepts of which it was partially composed, discharged their contents into the wound, the fluid being of a yellow, viscid character. The subsequent steps of the operation, including the disarticulation and the median section with the saw, were readily accomplished. Hemorrhage was tolerably free, but easily controlled. Interrupted silk sutures were used in uniting the mucous lining of the cheek, and continuous gut for the outside. A rubber drain was inserted at the most dependent point and allowed to remain for several days. Shock was well marked, but yielded promptly to proper measures. Some suppuration of the wound with febrile movement ensued, and retarded her recovery. She was discharged four weeks later.

Microscopical examination showed it to be an adeno-sarcoma that had probably developed centrally, expanding the plates of the bone, parts of the growth having undergone cystic degeneration.

CASE 2.—Osteo-Sarcoma of Right Upper Jaw. Excision. Recovery.

P. C., æt. 16, from Johnstown, Licking Co., Ohio, was sent by Dr. C. R. Lockwood, of that place. He had always enjoyed good health. In the early part of 1884 the right side of his face was seen to be getting larger, especially in the neighborhood of a defective upper molar tooth, from which the disease seemed to emanate. The growth was gradual until early in December, 1888. He entered the hospital February 8, 1889, at which time the right cheek was the seat of a prominence extending laterally from the molar bone to the side of the nose, and vertically from the angle of the mouth to the floor of the orbit. The latter was lifted up and the right eye slightly elevated. The hard palate was moderately depressed. There was no sign of fluetuation at any point.

Diagnosis.—A tumor of the upper jaw which had of late taken on such rapid growth that removal was deemed advisable.

Operation.—February 11, 1888. The entire right upper jaw was excised by the external flap method. An incision began opposite the inner angle of the eye, going vertically downward, encircling the wing of the nose and through the lip in the median line. Following again the natural fold between the cheek and the lower lid, it proceeded along the rim of the orbital floor as far as the molar bone. The flap could then be easily raised and the cheek was reflected from within outwards, thus securing a good exposure of the tumor. An incisor tooth was then extracted, and the saw was carried through the hard palate and the alveolus of the missing tooth, the nose being pushed out of the way. A section of the nasal process of the upper jaw and molar bone followed, the latter line of division being continuous with the spheno-maxillary fissure. The bones were of ivory-like hardness, so that their division was very tedious and difficult. Large pliers were forced into the notches made by the saw, thus loosening the growth, so that the pterygoid process could be broken and the soft palate cut away. The patient behaved badly under ether, a large quantity of which was required. The extreme hardness of he bones and the time consumed in dealing with them added to the delay. The wound was dusted with iodoform and stitched with gut inside and out, and no drain was used.

The shock was very profound. Evidently considerable concealed hemorrhage had occurred, for the patient vomited a large amount of clots and bloody fluid. The lower extremities were elevated, and injections of warm water and brandy were frequently given. Hypodermics of morphia and brandy were resorted to, dry heat was liberally applied, and after two hours of persistent effort vomiting ushered in reaction. Union was tolerable firm in 30 hours, and his convalescence was uneventful.

CASE 3.—Large Round-Celled Sarcoma of Right Upper Jaw. Excision. Recovery.

Mrs. M. A. T., æt. 55. Residence, Napoleon, Henry County, Ohio. In July, 1888, a swelling was first observed over the right molar bone which continued to grow until February 27, 1889, the date of her admission. Had had nose-bleed once a month before. Her health had always been good, and the growth was only slightly painful. The physical alterations, and the change in facial expression were about such as were seen in Case 2.

In view of the rapid development of the tumor excision was advised, and was done March 2, 1889. Aside from the fact that the incision was longer, the plan adopted was the same as that in Case 2. Much less difficulty was experienced in making the sections than in the former case, the bones being much softer. The wound having been carefully sutured, neat apposition was secured. External hemorrhage was very slight. One-third of a pint of clotted blood was vomited after the completion of the operation. Shock was hardly appreciable, and the pulse, although somewhat weak, was slow and regular. She passed a good night, and after the first week had no febrile disturbance. She was discharged March 24, three weeks after the operation.

CASE 4.—Sarcoma of Left Upper Jaw. Excision. Recovery.

Gertrude W., æt. 12; brought by Dr. Chambers, dentist, from Newark, Ohio. Eighteen months before, when cutting a molar tooth, she had observed that her left cheek was prominent. Two months before entering she had had a single nose-bleed. She was in good general health. The floor of the orbit was elevated by the growth, and the left side of the nose somewhat smaller and crowded forward. The roof of the mouth was depressed on the side involved. On November 6, 1889, the external flap operation was done in the usual manner. She had no unpleasant symptoms, and was discharged well in a fortnight.

CASE 5.—Recurrent (Myrtoid or Giant Cell) Sarcoma of Lower Jaw. Excision of Right Half. Recovery.

Mrs. A. W., æt. 36. Residence, Corning, Perry Co., Ohio. In 1875 she had undergone an operation for epulis of the lower jaw, in which several teeth with their alveoli were removed. Suspicions of recurrence were aroused early in November, 1889, and she entered the hospital three weeks later. A growth, central to the right maxilla had evidently started at, or near the angle, causing the bony plates to spread. It was growing forward on the body, and upward on the ramus. It had, of late, become painful, and excision was advised and done on November 30, in the usual manner. The anterior section was easily accomplished, as only the rim had been left when the epulis had been ablated. Care was observed in securing the vessels, but notwithstanding this fact, persistent and rather free secondary hemorrhage occurred during the night following the operation, which necessitated the removal of a few stitches, and reopening the wound. No vessels of appreciable size could be found, however, and Monsell's solution was applied. An opening was left for drainage and for the escape of sloughing particles. The bleeding never recurred, and the wound healed nicely by granulation. She was discharged in three weeks.

The original disease was epulis of a malignant type. The propriety of removal in cases of epulis is forcibly impressed upon us, and this had been thoroughly done by Dr. McGraw, of Detroit.

These cases, and one already reported in the *New York Medical Journal* of May 10, 1887, in which a large tumor and half of the lower jaw were excised, make six in all; three of the superior, and three of the inferior maxilla. If an apology be wanted for their presentation on this occasion, reference might be made to the fact that Dr. Deadrick, of Tennessee, in 1810, was the first to remove a portion of the lower jaw for tumor.

Reports of operations of the kind enumerated do not seem to be common nowadays. Whether it be on account of their infrequency, or because they are deemed unimportant, I can not say.

While an experience such as that indicated is not sufficiently broad to justify one in dogmatizing, I suspect that the larger proportion of these cases can be successfully handled without the preliminary delegation or tracheotomy; that the best way to *do* them is to get *through* with them as quickly as is compatible with thoroughness.

All the patients are free from recurrence to-day.

